# Dynamic of HIV-testing after arrival in France for migrants from sub-Saharan Africa: The role of both health and social care systems

**DOI:** 10.1371/journal.pone.0188751

**Published:** 2017-12-21

**Authors:** Frédérike Limousi, France Lert, Annabel Desgrées du Loû, Rosemary Dray-Spira, Nathalie Lydié, PARCOURS Study Group

**Affiliations:** 1 Santé publique France, Saint-Maurice, France; 2 Institut National de la Santé et de la Recherche Médicale (INSERM), CESP-U 1018, Villejuif, France; 3 CEPED, UMR Institut de Recherche pour le développement (IRD)-Université Paris Descartes, Sorbonne Paris Cité, Paris, France; 4 Sorbonne Universités, UPMC Univ Paris 06, Institut National de la Santé et de la Recherche Médicale (INSERM), Institut Pierre Louis d’Epidémiologie et de Santé Publique (IPLESP UMRS 1136), Paris, France; Katholieke Universiteit Leuven Rega Institute for Medical Research, BELGIUM

## Abstract

**Objective:**

HIV testing is an important tool in the management of the HIV epidemic among key populations. We aimed to explore the dynamic of first-time HIV testing in France for sub-Saharan migrants after their arrival.

**Methods:**

ANRS-Parcours is a retrospective life-event survey conducted from 2012 to 2013 in healthcare facilities in the Paris region, among 926 sub-Saharan HIV-infected migrants and 763 non-infected migrants. After describing the time to first HIV test in France and associated circumstances, we performed a discrete-time logistic regression to analyze the influence of socioeconomic position, contact with the healthcare system and sexual behaviors, on first-time HIV testing in France in migrants who arrived after 2000.

**Results:**

Median first-time HIV testing occurred during the second year spent in France for non-infected men and women in both groups, and during the first year for men of the HIV group. The probability of testing increased with hospitalization and pregnancy for women of both groups. For non-infected men unemployment and absence of a residence permit were associated with an increased probability of HIV testing [respectively, OR = 2.2 (1.2–4.1) and OR = 2.0 (1.1–3.5)]. Unemployment was also associated with an increased probability of first-time HIV-testing for women of the HIV group [OR: 1.7 (1.0–2.7)]. Occasional and multiple sexual relationships were associated with an increased probability of first-time testing only for HIV-infected women [OR: 2.2 (1.2–4.0) and OR = 2.4 (1.3–4.6)].

**Conclusion:**

Access to first HIV testing in France is promoted by contact with the health care system and is facilitated for unemployed and undocumented migrants after arrival.However, testing should be offered more systematically and repeated in order to reduce time between HIV infection and diagnosis, especially for deprived people which are particularly vulnerable regarding HIV infection.

## Introduction

HIV testing is an important tool in the prevention and management of the HIV epidemic. Early diagnosis and access to treatment are associated with reduced mortality and morbidity and limit the spread of the epidemic through the impact of early antiretroviral therapy on HIV transmission [1,2]. Testing is a major component of the UNAIDS plan to end the AIDS epidemic by 2030 [3]. In the European Union, an estimated 15% of individuals infected with HIV were undiagnosed in 2015 [4], and late presentation accounted for almost 47.9% of HIV diagnoses in 2010–2013 [5].

In France,as in other Western Europe countries, migrants, largely from sub-Saharan Africa (SSA), represent a significant proportion ofHIV cases (38% in 2015)[6]. They represent 40% of undiagnosed HIV-infected individuals. An estimated 9,300 non-French-national heterosexuals are unaware that they are HIV positive[7].

A large number of barriers to and drivers ofHIV testing have been identified in this population at an individual, social and structural level [8–10]. At the individual level, low risk perception and fear of stigma appear to be central determinants. Low social status and socioeconomic inequalitiesare also mentioned in the international literature as barriers to HIV testing and care [11]. Finally, health policy plays an essential role in ensuring access to HIV testing for migrants. The literature identified two main ways to promote HIV testing: population-wide approach and targeted interventions directed to most exposed populationsin partnerships with the communities[11]. Since the early 2000s, the national AIDS policy in France has targeted the migrant SSA population through dedicated programs and campaigns. Health services are encouraged to propose HIV testing to all adults and to repeat this offer each year for individuals exposed togreater risk. Since 2011, community-based testing programs using rapid tests have been launched in metropolitan areas to reach key populations including migrants and men who have sex with men. Despite this, the estimated time between HIV infection and diagnosis is still particularly high for people born outside France, with a mean of 53.5 months for non-French-national heterosexual men and 41.2 months for non-French-national heterosexual women [12].

We aimed to explore the time to first HIV test in France for sub-Saharan migrants, and to analyze factors associated with first-time testing for HIV-infected and non-infected people, using data from a large retrospective life-event survey.

## Methods

### Study design

The ANRS-Parcours study was conducted to examine how health trajectories and social and migratory paths are interconnected for migrants from sub-Saharan Africa living in France. This retrospective quantitative life-event survey was conducted from February 2012 to May 2013 in the greater Paris metropolitan area (Ile-de-France), among HIV-infected migrants born in sub-Saharan Africa receiving HIV care, and among non-infected migrants visiting primary-care centers. Recruitment took place at facilities randomly selected from an exhaustive list of HIV outpatient hospital clinics (N = 24) and general practice medical centers (N = 30). Patients were eligible if they were born in sub-Saharan Africa, aged 18 to 59, and either diagnosed with HIV infection at least 3 months previously(for the HIV group), or not diagnosedwith HIV (for the primary care group). Doctors asked all eligible patients—except those with major cognitive or health impairments—to participate and obtained their written consent.

A trained interviewer administered a face-to-face standardized life-event history questionnaire to each participant. It consisted of a list of questions associated with a biographical grid, which allowed identifying and dating (per year) events of interest in respondent’s life, from birth to the date of the survey; and a book of thematic modules, aimed at describing in-depth the events previously identified(see S1 and S2 Appendices). Professional interpreters were available upon request. Information collected included socio-demographic characteristics, conditions of migration and life in France, relational, and reproductive history, and healthcare pathways, including HIV testing. Clinical and laboratory information was documented from medical records. All information was collected anonymously.

Participants received a 15€ voucher. The Advisory Committee on Data Collection in Health Research and the French Data Protection Authority approved this project. The complete survey protocol is registered at Clinical-trials.gov (NCT02566148).

### Population of interest

Only persons arriving after 2000 were included because non-national populations became a priority of the national plan against HIV from that year on. Individuals who had never had sexual relationships before arrival were excluded, in order to only consider sexually active people who were likely to benefit from testing from the moment of arrival.

In the HIV group, we excluded persons who were already diagnosed before arrival in France.

### Outcomes and variables of interest

Participants were asked if they had already been tested for HIV and what the date, results, and surrounding circumstances were for each previous test. Circumstances of testing were then classified into 3 categories: voluntary testing (just to know, HIV infection of the partner, risky behaviors,desire to stop using condoms, partner’s request that respondent have a test); medical reason(part of a health check-up, physician request, illness or hospitalization), and systematic screening(prenatal or prenuptial testing, blood donation, residence permit application, insurance, travel or mortgage requirements). Based on this information, we identified the dynamic of participants’ first HIV test after arrival in France (i.e. time to test and circumstances). The HIV group comprised those already infected before arrival (i.e., diagnosis occurred at first HIV test in France), and those infected with HIVany time after arrival (i.e., diagnosis not necessarily occurring at first HIV test in France).

Several categories of determinants of the first test were consideredfor each person and comprised both fixed and time-dependent variables. Fixed variables included educational level at arrival (none or primary / secondary / post-secondary), HIV prevalence in country of origin in 2013 [13] (< 1% / 1–3% / ≥ 3%), and reason for migration (work,joinfamily or study / feeling threatened in their home country / medical reasons). Time-dependent variables includedthe following:

- Socioeconomic and administrative position: temporary housing if participants had been living in the same premises for less than a year, unemployment (neither declared nor undeclared job), absence of residence permit and absence ofhealth coverage.
- Contact with the healthcare system: hospitalization and pregnancy
- Previous sexual relationships taking into account all encounters before the year considered (only stable relationships / casual or transactional relationships / multiple relationships). Multiplerelationships were defined as declaring at least two encounters of any kind for the same year. Categories were exclusive and hierarchic. If a participant declared casual or transactional relationships and multiplerelationships in the past, he or she was classified in the category “multiplerelationships”.

The unit of analysis for all time-dependent variables was the calendar year. All years between arrival in France and the first HIV test (or moment of the survey if not yet tested) were included. The objective was to determine the influence of these determinants on the probability of being tested for each year included.

### Statistical analysis

Characteristics of the HIV and primary care groups were compared using chi-squared tests or t-tests as appropriate for quantitative variables. All analyses were then stratified by group and by sex.

We performed a Kaplan-Meier analysis to describe the time between arrival in France and first HIV test, and used a discrete-time logistic regression to analyze factors associated with the probability of HIV testingfor each year included, taking into account both fixed and time-dependent variables. Data were weighted according to each individual’s probability of inclusion in the survey. Analyses were conducted using STATA 13.1 (Stata Corp., College Station, Texas, USA). Data are available in S1 Dataset.

## Results

### Study population

The ANRS-Parcours study included 763 people in the primary care group and 926 in the HIV group. In the primary care group, 196 men and 205 women met the study criteria of arrival in France after 2000 and sexually active before arrival. In the HIV group, a total of 484 people met these two criteria. Among them, 134 men and 258 women had not been diagnosed before their arrival. (Table 1)

**Table 1:** Characteristics of participants in the primary-care and HIV groupsby sex. ANRS-Parcours study.

		Men n(weighted %)			Women n(weighted %)		
		Primary-care (N = 196)	HIV (N = 134)	p	Primary-care (N = 205)	HIV (N = 258)	p
**Median time since arrival in France (IQR)**		2 (1–8)	7 (3–9)	< 0.01	3 (1–7)	7 (4–10)	< 0.01
**Characteristics at arrival**							
**Median age (IQR)**		31.5 (26–38)	35 (29–40)	0.01	30 (25–38)	31 (26–36)	0.24
**Educational level**				0.06			0.36
	none/primary	57 (27.7)	36 (22.6)		40 (17.0)	58 (20.0)	
	secondary	102 (56.9)	65 (49.2)		126 (58.0)	155 (62.1)	
	post-secondary	37 (15.4)	33 (28.2)		39 (25.0)	45 (17.9)	
**HIV prevalence in country of birth**^**1**^				< 0.01			< 0.01
	< 1%	75 (35.1)	25 (16.7)		48 (27.6)	33 (10.6)	
	1–3%	98 (55.1)	66 (49.6)		112 (55.9)	143 (56.6)	
	≥ 3%	23 (9.8)	43 (33.7)		45 (16.6)	82 (32.8)	
**Reason for coming to France**				0.36			< 0.01
	work/family/studies	133 (64.1)	100 (71.8)		126 (63.0)	205 (78.5)	
	feeling threatened in their country	53 (32.7)	28 (23.3)		64 (31.1)	32 (12.1)	
	medical reasons	10 (3.3)	6 (4.9)		15 (5.9)	21 (9.4)	
**Previous relationship**				0.05			0.03
	only stable partnerships	25 (11.1)	17 (15.4)		86 (41.9)	80 (35.1)	
	casual/transactional partnerships	76 (41.2)	38 (24.9)		69 (38.9)	91 (32.7)	
	Multiple partnerships	95 (47.7)	79 (59.7)		50 (19.2)	87 (32.3)	
**Situations experienced at least once since arrival**							
**Socioeconomic conditions**							
	temporary housing	88 (43.2)	57 (41.2)	0.77	82 (34.2)	93 (34.4)	0.98
	unemployment	105 (52.8)	60 (40.6)	0.08	163 (76.9)	151 (59.7)	< 0.01
	absence of residence permit	121 (54.0)	82 (66.2)	0.07	116 (50.8)	149 (55.3)	0.45
**Health and healthcare access**							
	Pregnancy^2^	59 (33.3)	27 (23.4)	0.13	92 (49.5)	72 (29.5)	< 0.01
	hospitalization	21 (11.5)	38 (30.3)	< 0.01	25 (11.0)	47 (21.0)	0.02
	absence of health coverage	112 (52.7)	63 (48.3)	0.53	103 (44.5)	96 (35.3)	0.11

^1^ Prevalence estimation in 2013, 15–49 years

^2^For men: partner’s pregnancy

The men in the HIV group were older than those in the primary care group (median 35 years versus 31.5 years). They were more likely to be from a country with an HIV prevalence ≥ 3% (34% versus 10%), had been in France longer(7 years versus 2 years), and had been hospitalized more often since their arrival (30% versus 12%). No significant differences were observed between the groups for other characteristics.

At the time of the survey, the women in the HIV group had spent more time in France than those in the primary care group (7 years versus 3 years). They were more likely to come from a country with an HIV prevalence ≥ 3%(33% versus 17%) and less likely to have migrated because they felt threatened in their country of birth (12% versus 31%). In the HIV group, 32% of women had already had multiple relationships, compared withonly 19% in the primary care group. In the HIV group,fewer womenhad experienced at least one period of unemployment (60% versus 77%) and fewerhad become pregnant since their arrival (30% versus 50%).

### Timebefore first test, circumstances of testing, and associated factors

**Fig 1:**
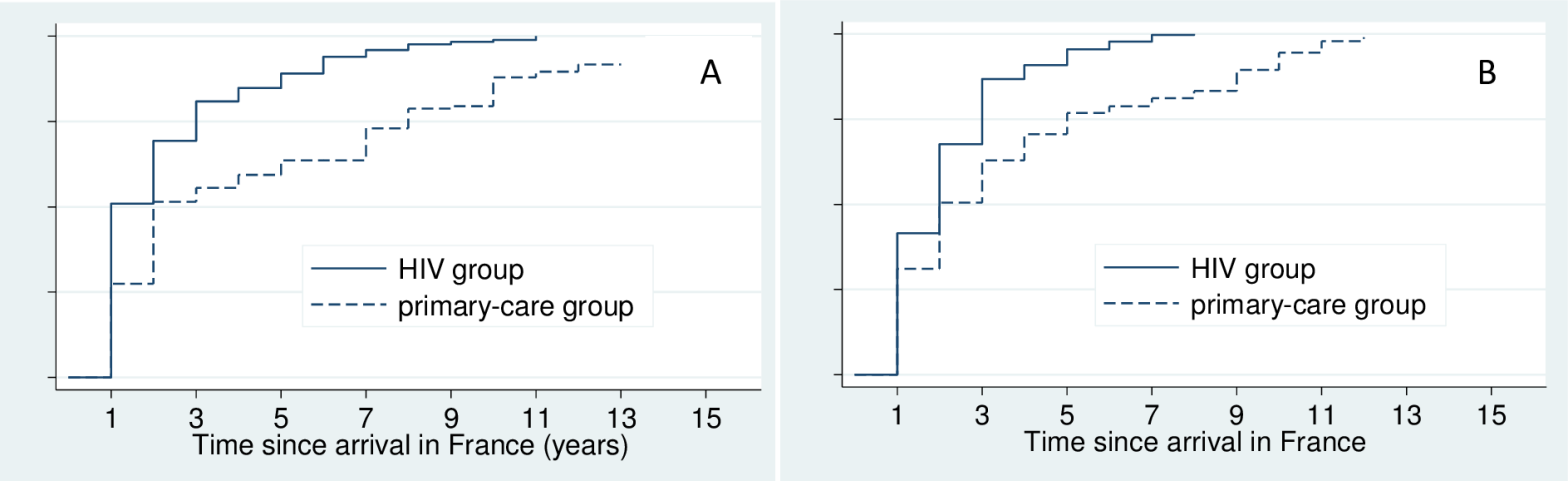
Kaplan Meier estimate of the probability of HIV screening among men (1.A) and women(1.B) over time since arrival in France, by group. ANRS-Parcours study.

For men, first-time HIV testing in France occurred in median during the first year in the country forthe HIV-group (IQR: 1–3) and during the second year for the primary-care group (IQR: 1–8)(Fig 1). First-time testing occurredformedical reasons in63% and 69% of the men in the HIV and primary care groups, respectively,generally as part of a health check-up.In the HIV group, 21% of men had been tested because they were ill versus 2% in the primary-care group. In both groups, voluntary testing was reported by less than30% of men (Table 2).In the HIV group, 87% of men were diagnosed HIV positiveat their first test in France.

**Table 2:** Circumstances of test among participants in the primary-care and HIV groupsby sex.

	Menn (weighted %)		Womenn (weighted %)	
	Primary-care group (N = 135)	HIV group (N = 134)	Primary-care group (N = 162)	HIV group (N = 258)
Circumstances of the test				
**Medical reasons**	**92 (68.8)**	**87 (63.4)**	**86 (50.8)**	**137 (54.5)**
General health check-up	52 (38.5)	33 (26.2)	37 (21.8)	51 (18.2)
Physician request	36 (27.7)	14 (8.8)	39 (23.7)	32 (11.8)
Illness	3 (2.4)	28 (20.8)	4 (1.4)	37 (17.3)
Hospitalization	1 (0.2)	12 (7.7)	6 (3.9)	17 (7.2)
**Systematic screening**	**5 (3.2)**	**6 (5.7)**	**42 (32.2)**	**70 (27.1)**
Pregnancy related	1 (0.9)	4 (4.0)	34 (25.7)	61 (23.8)
Prenuptial test	-	2 (1.7)	5 (3.6)	4 (1.7)
Residence permit, insurance, travel or mortgage requirements	4 (2.3)	-	3 (2.9)	4 (1.1)
Blood donation	-	-	-	1 (0.5)
**Voluntary testing**	**36 (25.9)**	**40 (29.5)**	**30 (15.4)**	**46 (16.2)**
Just to know	31 (23.1)	27 (19.6)	27 (14.0)	44 (15.0)
HIV-infected partner	-	6 (4.2)	-	-
At-risk situation	2 (1.6)	6 (4.7)	1 (0.7)	-
Desire to stop using condoms	-	1 (1.0)	2 (0.7)	1 (1.0)
Partner asked participant to stop using condoms	2 (1.2)	-	-	1 (0.2)
**Other reason**	**3 (2.1)**	**1 (1.5)**	**4 (1.7)**	**5 (2.2)**

For men in both groups, educational level, housing situation, previous sexual relations, and healthcoverage were not significantly associated with HIV testing (Table 3). In the HIV group, the probability of testing increased with age and was significantly associated with hospitalization[odds ratio (95% confidence interval); OR = 9.8 (2.5–38.6)].Men from countries with an HIV prevalence ≥ 3% and men who came to France for medical reasons were tested earlier after their arrival [respectively, OR = 2.1 (0.9–4.7) and OR = 11.7 (1.1–120.6)]. The absence ofa residence permit was associated with a decreased probability of being tested [OR = 0.4 (0.2–0.9)]. In the primary care group, theprobability of testing decreased in the third year spent in France compared with the first year [OR = 0.3 (0.1–0.9)] while unemployment and absence of a residence permit were associated with an increased probability of HIV testing[respectively, OR = 2.2 (1.2–4.1) and OR = 2.0 (1.1–3.5)].

**Table 3:** Factors associated with first HIV test in France among men coming from sub-Saharan Africa, in primary-care group and in HIV group (mixed-effects logistic regression models). ANRS-Parcours study.

		Primary-care group (N = 196)					HIV group (N = 134)				
		Person- Years	HIV test	OR	[95% CI]	p-value	Person- Years	HIV test	OR	[95% CI]	p-value
**Characteristics at arrival**						** **					
**Educational level**						0,07					0.38
	None/primary	225	29	ref			82	36	ref		
	Secondary	270	79	2.3	[1.1–4.6]		141	65	1.5	[0.7–3.1]	
	Post-secondary	99	27	1.9	[0.4–4.8]		92	33	0.9	[0.4–2.0]	
**HIV prevalence in country of birth**						0,68					< 0.01
	< 1%	261	53	ref			66	25	ref		
	1–3%	285	66	0.8	[0.4–1.4]		170	66	0.6	[0.3–1.1]	
	≥ 3%	48	16	1.0	[0.4–2.2]		79	43	2.1	[0.9–4.7]	
**Reasons for coming in France**						0,85					0.09
	Family/work/study	426	88	ref			258	100	ref		
	Feeling threatened in their country	145	38	0.9	[0.5–1.8]		50	28	1.4	[0.6–3.2]	
	Medical reasons	23	9	1.2	[0.4–3.3]		7	6	11.7	[1.1–120.6]	
**Time-varying variables**						** **					
**Age (years)**						0.74					< 0.01
	16–30	186	47	ref			98	27	ref		
	30–34	130	33	0.8	[0.4–1.5]		62	31	2.5	[1.2–5.0]	
	≥ 35	278	55	0.8	[0.4–1.5]		155	76	5.2	[2.0–13.2]	
**Time in France (years)**						0.03					0.46
	1	196	61	ref			134	69	ref		
	2	121	35	1.4	[0.8–2.7]		65	25	0.7	[0.3–1.7]	
	3	73	9	0.3	[0.1–0.9]		40	16	0.6	[0.2–1.7]	
	≥ 4	205	30	0.8	[0.4–1.5]		76	24	0.6	[0.3–1.2]	
**Previous relationships**						0.24					0.78
	Only stable partnerships	111	13	ref			41	16	ref		
	Casual/transactional partnerships	246	49	1.0	[0.4–2.5]		81	38	1.3	[0.6–3.0]	
	Multiple partnerships	237	73	1.6	[0.7–3.9]		193	80	1.1	[0.5–2.6]	
**Housing**						0.13					0.31
	Stable	456	94	ref			235	91	ref		
	Temporary	138	41	1.6	[0.9–2.8]		80	43	1.5	[0.7–3.2]	
**Activity**						0.01					0.69
	Work or study	416	76	ref			221	85	ref		
	Unemployed	178	59	2.2	[1.2–4.1]		94	49	0.9	[0.4–1.7]	
**Residence permit**						0.03					0.03
	Yes	317	61	ref			153	76	ref		
	No	277	74	2.0	[1.1–3.5]		162	58	0.4	[0.2–0.9]	
**Health insurance**						0.07					0.61
	No	182	47	ref			104	42	ref		
	Yes	412	88	1.8	[0.9–3.6]		211	92	0.8	[0.3–1.9]	
**Hospitalization**						0.73					< 0.01
	No	573	128	ref			278	102	ref		
	Yes	21	7	1.2	[0.4–4.4]		37	32	9.8	[2.5–38.6]	

For women, first-time HIV testing in France occurred in median during the second year spent in France for both groups (IQR: 1–3 and IQR: 1–5 for the HIV and primary-care groups, respectively) (Fig 1). First-time testing was conducted for medical reasons for 55% and 51% of women, respectively. In the HIV group, 17% of women were tested because they were ill versus 1% in the primary-care group. In both groups, testing was conducted through systematic screening in approximately 30% of cases, most often during pregnancy. Voluntary testing was reported by almost 15% of women in both groups (Table 2). In the HIV group, 93% of women were diagnosed HIV positive at their first test in France.

For women in both groups, testing occurred earlier after arrival in France among individuals from countries with an HIV prevalence ≥ 3% and among those who came to France for medical reasons (Table 4). In both groups the probability of testing was strongly associated withhospitalization [OR = 22.3 (6.2–79.8) and OR = 5.6 (1.8–17.6) in the HIV and primary-care groups, respectively] and with pregnancy [OR = 8.4 (4.1–17.2) and OR = 7.3 (3.6–14.8) in the HIV and primary-care groups, respectively]. As was observed among men, educational level and housing situation were not significantly associated with testing in either group. In the HIV group, the probability of testing was higher among women who had casual or transactional relationships[OR = 2.2 (1.2–4.0)] and among women who had multiplepartners [OR = 2.4 (1.3–4.6)],compared with women who had only had stable relationships. Unemployment and health insurance were associated with an increased probability of HIV testing[OR = 1.7 (1.0–2.7) and OR = 2.7 (1.5–5.0)].

**Table 4:** Factors associated with first HIV test in France among women coming from sub-Saharan Africa, in primary-care group and in HIV group (mixed-effects logistic regression models). ANRS-Parcours study.

		Primary-care group (N = 205)					HIV group (N = 258)				
		Person- Years	First HIV test	OR	[95% CI]	p-value	Person- Years	First HIV test	OR	[95% CI]	p-value
**Characteristics at arrival**											
**Educational level**						0.05					0.36
	None/primary	99	28	ref			148	58	ref		
	Secondary	386	98	0.7	[0.3–1.7]		313	155	1.6	[0.8–3.0]	
	Post-secondary	92	36	1.3	[0.5–3.3]		89	45	1.2	[0.5–2.8]	
**HIV prevalence in country of birth**						0.02					0.04
	< 1%	175	36	ref			87	33	ref		
	1–3%	285	92	2.2	[1.2–3.9]		317	143	1.8	[0.9–3.5]	
	≥ 3%	117	34	2.1	[1.0–4.3]		146	82	2.6	[1.2–5.5]	
**Reasons for coming in France**						0.06					0.05
	Family/work/study	409	97	ref			445	205	ref		
	Feeling threatened in their country	137	53	1.5	[0.9–2.7]		77	32	0.9	[0.5–1.6]	
	Medical reasons	31	12	3.1	[1.0–9.3]		28	21	4.1	[1.3–13.0]	
**Time-varying variables**											
**Age (years)**						0.53					0.62
	16–30	244	63	ref			229	96	ref		
	30–34	116	31	1.3	[0.6–2.7]		140	76	1.3	[0.7–2.4]	
	≥ 35	217	68	1.4	[0.8–2.3]		181	86	1.0	[0.5–1.7]	
**Time in France (years)**						0.25					0.08
	1	205	73	ref			258	112	ref		
	2	117	34	0.8	[0.4–1.5]		146	74	1.3	[0.8–2.2]	
	3	68	17	0.9	[0.4–2.2]		72	39	2.3	[1.1–4.8]	
	≥ 4	187	38	0.5	[0.3–1.0]		74	33	2.0	[0.9–4.2]	
**Previous relationships**						0.99					< 0.01
	Only stable partnerships	247	63	ref			165	69	ref		
	Casual/transactional partnerships	207	57	1.0	[0.6–1.8]		196	97	2.2	[1.2–4.0]	
	Multiple partnerships	123	42	1.0	[0.5–1.9]		189	92	2.4	[1.3–4.6]	
**Housing**						0.47					0.41
	Stable	457	117	ref			417	189	ref		
	Temporary	120	45	1.3	[0.6–2.8]		133	69	1.2	[0.7–2.1]	
**Activity**						0.48					0.04
	Work or study	267	68	ref			315	139	ref		
	Unemployed	310	94	1.2	[0.7–2.0]		235	119	1.7	[1.0–2.7]	
**Residence permit**						0.16					0.15
	Yes	345	101	ref			258	144	ref		
	No	232	61	0.6	[0.3–1.2]		292	114	0.7	[0.4–1.1]	
**Health insurance**						0.73					< 0.01
	No	152	45	ref			157	46	ref		
	Yes	425	117	1.1	[0.6–2.3]		393	212	2.7	[1.5–5.0]	
**Hospitalization**						< 0.01					< 0.01
	No	557	150	ref			502	215	ref		
	Yes	20	12	5.6	[1.8–17.6]		48	43	22.3	[6.2–79.8]	
**Pregnancy**		** **				< 0.01					< 0.01
	No	482	114	ref			480	203	Ref		
	Yes	95	48	7.3	[3.6–14.8]		70	55	8.4	[4.1–17.2]	

## Discussion

### Statement of principal findings

For men in the primary-care group and women of both groups, first-time HIV testing in France occurred in median during the second year after arrival. For men in the HIV group it occurred earlier, in median during the first year. First-time testing was conducted more often for medical reasons. Unemployment and contact with the healthcare system were facilitators to HIV testing. Multipleor occasional relationships were associated with an increased probability of testing only for women in the HIV group.For women and men in the HIV group, the first HIV test in France was positive in most cases. First-time testingbecause of illness was more frequent in the HIV group than in the primary-care group.

### Strengths and weaknesses

Prospectively monitoring a cohort of migrants arriving in the country, sometimes clandestinely, is impossible. The biographical design of this study therefore offers a viable alternative as it retrospectively reconstructs their trajectories. Recall bias was minimized by using a life-event questionnaire based on “noteworthy” events,which contextualized events in relation to one another. With this approach, however, it was not possible to collect certain kinds of longitudinal information, including knowledge of and attitudes towards HIV, elements which may change over time and which are known to influence testing behaviors[8–10]. Moreover, as the unit of time used was one calendar year, no timeline could be established for two events occurring in the same year.

Another limitation of the study is that it was only performed in the Ile-de-France area (Paris and some suburbs) and therefore it may not be representative of all migrants from SSA in France. However, 60% of themreside in this region [14].Furthermore, even though the study population was restricted to healthcare users, the recruitment in different types of healthcare structures provided us with a large and diverse sample. Moreover, in France, migrants can access medical care for free after spending 3 months in the country, regardless of their legal status.

### Comparison with existing literature

There is little comparative data in the literature concerning factors that influence HIV testing behaviorsjust after migrating. In the United Kingdom, a study conducted among key informants in the field of migrant health suggested that HIV testing occurred within 3 years after arrival through health services (especially during pregnancy for women) or through social services [15]. In France, the median time between arrival and diagnosis was estimated to be 2 years for men and 1 year for women according to a study conducted among HIV-positive patients in 2011 [16], which isin line with our results.

In our study, unstable living conditions had a different impact on HIV testing in men and women. For men in the primary care group, the lack of a residency permit and unemployment were associated with an increased probability of testing.In previous studies in Western countries, high levels of unemployment and poverty in migrants and ethnic minorities, low social status and inequalities are mentioned as barriers to HIV testing and care [11]. Issues around legal status were also cited as a barrier to testing [8]. In Europe, uninsured and undocumented migrants are often reluctant to test because of restricted access to healthcare services [10]. Unlike in other Western countries, HIV diagnosis in France could lead to obtain a residence permit for medical reasons since 1998 and the fear of deportation might be alleviated.Provision of free health care for uninsured or deprived people, among whom many recent migrants, through dedicated services and NGOs might explain the observed association between poor social status and earlier testing.In such services, HIV testing is often offered on a routine basis at first contact.

In a more general way, physicians are more likely to suggest HIV testing forunderprivileged patients, for example to patients without health coverage or those who benefit fromspecific state-provided medical assistance for migrants (called AME in France)than for those with health coverage [17].

Unlike men in theprimary care group, having a residency permit was associated with HIV testing for men in the HIV group. However, this observed association might be artificial since the positive HIV test and legal permit might occur the same year. Actually, HIV diagnosis allows undocumented migrants to benefit a residence permit for medical reasons but the questionnaire was not designed to describe the sequence of events occurring the same calendar year.The role of administrative and economic deprivationwas less clear among women.The probability of testing was higher during periods of unemployment only for women in the HIV group and health coverage was associated with higher rates of testing among women in the HIV group. Although testing is anonymous and free in France, thisresultmight be the consequenceof greater awareness of the health system and testing centers by migrants withhealth coverage [18].

After the first year spent in France, the probability of testing decreased significantly for men. HIV infection in sub-Saharan migrants is often viewed as acquired before migration while we have estimated in a previous study that 44% and 30%, respectively, of HIV-positive men and women born in SSA, became infected in France [19]. Testing is often offered during the early period in France but physicians are less likely to propose testing and to repeat the proposal over time since the risk of infection in France is underestimated [17].

The first years spent in France are a period of vulnerability in which most migrants experience unstable living conditionsand we have shown in a previous paper that poor living conditions are associated with HIV infection after migration, especially in women [20].

In women from both groups, HIV testing had mostly occurred following contact with the health system, with a very strong association between HIV testing and hospitalization, pregnancy, and coming to France for medical reasons. Approximately one quarter of the women from both groups wasHIV tested for the first time in Franceduring pregnancy.In France, prenatal HIV testing has been systematically offered to pregnant women during their first prenatal consultation since 1993. The strong association between testing and contact with the health system through hospitalization was also very clear for men in the HIV group, suggesting that they already presented symptoms of the disease when they went to be tested. Indeed, 21% of men in that group declared that HIV testing was motivated by illness. For men in the primary care group, hospitalization was not significantly related to HIV testing. This is consistent with a study of newly diagnosed HIV-positive patients in France which showed that missed opportunities for testing were commonplace in 2010, despite the fact that any contact with the healthcare system should be an opportunity to perform a test in people from at-risk groups [21].

Given the absence of any association between sexual behaviors and HIV testing in our SSA migrant study sample, and the low proportion of voluntary testing, perception of risk did not seem to bea major determinant in going for first-timeHIV testing in France, particularly for men. This result is consistent with previous studies which showed that HIV testing in migrants from sub-Saharan Africais not influenced very much by risky behavior[22]. A study conducted in the United Kingdom examining missed opportunities for HIV testing among newly diagnosed SSA migrants, highlighted that the main factor preventing participants from testing for HIV earlier,was that they did not consider the possibility that they might be HIV-positive (69.9% of respondents).Conversely, 59% believed they would have tested earlier if someone had told them they were at risk [23]. For women in the HIV group of our study, it seems that HIV testing was more influenced by the perception of sexual risk. In fact, HIV testing was more frequent among women who had previously had casual partnerships and/or multiple partnerships,than in those who had had only stable relationships. Gender-specific differences in perception of risk were observed in a study conducted in 2005 among migrants from SSA in the Ile-de-France region. In that study, women who had had at least two partners in their lives had a higher perception of the risk of contracting HIV than women who had had only one partner (21.5% versus 8.9%). This difference was not observed among men (9.2% versus 8.9%) [24]. In our study, for men in the primary care group, no association was found between the prevalence of HIV in their country of birth and HIV testing, whereas for women in both groups and men in the HIV group, a prevalence ≥3% was associated with earlier HIV testing. This might be explained by differences in knowledge and socio-cultural perceptions related to HIV policies in African countries most affected by the HIV epidemic[25,26].

### Implications for clinicians and policymakers

Most of the migrants were tested for HIV in the two years following their arrival in France, most often at the initiative of health workers, who therefore play a major role in testing.Despite the fact that specific settingsdedicated to undocumented migrantsare already playing an important role,the low levels of voluntary testing and absence of any association between sexual behavior and HIV testing highlighted in this study,suggest that health workers need to be more proactive and take all opportunities to encourage testing, both during hospitalization and in primary care. Pregnancy, which remains the main opportunity for HIV testing for women, should also be an opportunity to offer testing to their partners, thereby increase testing rates in men. Moreover, screening should be offered more routinely especially to healthy people who are not in contact with the health system. All existing measures should be strengthened combining provider-initiated testing, outreach testing by community organisations, provision of free self-tests and communication on testing and care opportunities in France, in particular mobile community-based programs promoting and offering rapid HIV testing in the street.It is not only essential to conduct HIV testing immediately upon a migrant’s arrival, but also to maintain easy access to testing and repeat testing for people who have lived in France for several years, especially in the context of deprivation which is associated with a greater risk of infection [20] and a higher HIV prevalence [27], in order to better control the epidemic and improve the prognosis of infected people.

## Supporting information

S1 Appendix Questionnaire. (DOCX)

S2 Appendix Biographical grid. (PDF)

S1 Dataset Data. (DTA)
