# Supplementary material for: Dynamic of HIV-testing after arrival in France for migrants from sub-Saharan Africa: The role of both health and social care systems
Source: PLoS One. 2017 Dec 21;12(12):e0188751. doi: 10.1371/journal.pone.0188751 (PMC5739385; doi:10.1371/journal.pone.0188751)
Supplement: S1 Appendix — (DOCX) [file pone.0188751.s001.docx]

# Questionnaire Patient PARCOURS

Chap1

**- T1**

- Conducted by researchers and designed together with associations of patients and physicians
- Addresses health and living conditions of people born in Africa and now living in France.

**- T2**

- Questions about the highlights of your life from your childhood until now
- Responses are recorded on the computer and the biographical paper grid (show it) helps to identify the different stages of your life
- All data collected is completely anonymous and confidential (will not be shared with the medical team or anyone else)

**Q3. You have been informed that you were seropositive for HIV-1 at least three months ago?**

1. Yes

2. No, I'm not seropositive for HIV1

3. I am HIV-positive but only for HIV2

4. I was informed of my seropositivity for HIV-1 less than three months ago

**Q3. You have been informed that you had chronic hepatitis B at least three months ago?**

1. Yes

2. No, I do not have hepatitis B

3. I was informed I had hepatitis B less than three months ago

**Q7. For a start and to set a timeframe, can you tell me what year you were born (e)?**

**Q7a. Or can you tell me your age? | ___ | ___ |**

**We first are going to note on this grid the milestones in your life since your childhood then we will further detail some of these events.**

Chap2

**We'll start by talking about the countries you have lived in for at least a year, starting from when you were born until now ...**

**- When you were born, in what country were you living?**

**- How long (until what age) did you stay in this country?**

**- And then, in what country did you live?**

**- How long (until what age) did you stay in this country?**

**We are now going to review in detail all the different dwellings you lived in for more than a year IN FRANCE. Some of these dwellings may be in the same city.**

**- When you arrived in France, did you live more than a year in the same dwelling or have you frequently changed dwellings?**

**- When you arrived in France, the dwelling where you lived was in which administrative region/French “département”?**

**- When you settled in this dwelling:**

**1. It was your own home;**

**2. You were hosted by your family (with or without financial support);**

**3. You were hosted by another person (s) close to you (with or without financial support);**

**4. Other to specify.**

**- How long did you stay in this dwelling?**

**- And then, did you live more than a year in the same dwelling or have you frequently changed dwellings?**

**- In which administrative region/French “département” was this second home located?**

**During this time when you frequently changed dwellings, did you happen to:**

*** Be hosted by associations or institutions?**

*** Be successively hosted by some friends or family members?**

*** Sleep in a squat?**

*** Sleep on the street?**

**We are now going to talk about the different periods of activities since your childhood: school, studies, vocational training, work, home or unemployment, etc. Let's talk about the activities that have lasted at least a year.**

**- What were you doing when you were 6 years old? What was your main activity?**

**How long did you continue to (*adapt)* to go to school / help at home ... ?**

**- And then what did you do? And until when?**

**In this activity you were ...?**

1. Manager(middle management)

2. Employee or worker

3. Boss, employer

4. Self-employed (without employees)

5. Apprentice, trainee

6. Caregiver

**- What type of contract did you have?**

1. CDI (Contract of Indeterminate Duration)

2. CDD (Contract of Determinate Duration)

3 - No contract

**- And then, what did you do? And until when?**

**During your entire professional life, were there times when, due to your activity, you had to travel a lot abroad or to another city?**

**Q8. To summarise your studies:**

1. You’ve never been to school

2. You’ve only been to Coranic School

3. You’ve been to school

**Q8a. What is the last class you took?** *97. School system does not match*

| **01** : Pré-scolaire (maternelle)  **Elémentaire-Primaire**  **02** : Cours d’Initiation CI  **03** : Cours Préparatoire CP  **04** : Cours Elémentaire 1 CE1  **05** : Cours Elémentaire 2 CE2  **06** : Cours Moyen 1 CM1  **07** : Cours Moyen 2 CM2 | **Secondaire - Collège**  **08** : 6^ème^  **09** : 5^ème^  **10** : 4^ème^ / CAP 1  **11** : 3^ème^  / CAP 1    **Secondaire - Lycée**  **12** : 2^nde^ / BEP 1  **13** : 1^ère^ / BEP 2  **14** : Terminale | **Supérieur**  **15** : 1^ère^ année (DEUG 1, L1 ou équivalent) / BTS 1  **16** : 2^ème^ année (DEUG 2, L2 ou équivalent) / BTS 2  **17** : 3^ème^ année (Licence, L3 ou équivalent)  **18** : 4^ème^ année (Maîtrise, M1 ou équivalent)  **19** : 5^ème^ année (DESS, DEA, M2 ou équivalent)  **20** : 6^ème^ année et au-delà (doctorat…) |
| --- | --- | --- |

**Q8b. From your entry to school, how many years have you attended school or been studying?**

**Q9. Do you have a degree?**

1. Yes

2. No

**Q9d. What is your highest degree?**

**Can we see together where your financial resources have been coming from at different times of your life?**

**• At what age did you have other resources than those of your parents?**

**• Where did these resources come from?**

1. Your own activity;

2. The activity of your spouse/partner

3. Activities of other family members;

4. State grants, specify (scholarship, family allowance, disability or invalidity allowance...);

5. No resources.

**• How long did you have this type of resource?**

**• And then what did you have as resources? And until when?**

Chap3

**We will now discuss the highlights of your emotional and family life: relationships or marriages, children and pregnancy that you or your partner have had.**

**This study concerns 3000 people. Researchers need to anticipate all situations. Some questions may not apply to you and you just have to say so if this is the case.**

**Q10. First, can you tell me, in total, how many men you have ever had sex with (within marriage or not)?**

**Q10a. Would you say ...**

1. Less than 5

2. 5 to 10

3. 11 to 20

4. More than 20

**Q11. And how many women?**

**Q11a. Would you say ...**

1. Less than 5

2. 5 to 10

3. 11 to 20

4. More than 20

Chap 3.1

Let's talk about the RELATIONSHIPS you have been and that have lasted at least a year, while being married or not, including people with whom you are now separated or who died, whether these relationships are with women/men if this is the case for you.

Q11bis. Have you been in any relationship?

1. Yes

2. No

*** Let’s talk about the first person you’ve been in a relationship with for at least a year. Can you give me a name for this person, this will help us to conduct the interview?**

**When did your relationship with "*name*” start?**

**Are you still together?**

Separation, divorce, death

**When did that relationship end? How did it end?**

**Have you had another relationship of more than a year?**

**Lets be more specific about each person that we have talked about.**

**100. So you have been in a relationship during more than one year with.......... people.**

**- “Name” is:**

1. A man

2. A woman

**101. In what country "Name" is/was he/she born?**

**102. Is/was he/she younger/older than you?**

1. Younger

2. Older

3. 1bout the same age (+/- 2 years)

**- And how many years older/younger?**

**103. What was her/his education level early in your relationship?**

1. Unschooled

2. Primary school

3. Secondary education (college, high school)

4. Higher education

**104. Were/are you married with "Name"?**

1. Yes

2. No

3. No but “pacsé(e)”

**105. Do you live together/have you lived together?**

1. Yes, during our whole relationship

2. Yes but not all the time

3. No, never

**106. Was there a period or periods in your relationship when you were not both living in the same country?**

1. Yes

2. No

**107. Did "name" have other stable partners during your relationship?**

1. Yes

2. No

Chap3.2

We will now discuss your relationships that were shorter, that is to say they lasted less than a year or were casual, whether with women/men if this is the case for you. "

Q12. Have you had short relationships (less than a year)?

1. Yes

2. No

**Can we see together at what time in your life these relationships took place?**

Chap3.3

*** Now can you tell me if you have ever been in a relationship that you have accepted, but that you did not really want, in order to find shelter, clothes, food, papers etc.. ?**

**Can you tell me at what times in your life this has happened?**

*** Have you ever had sex for money?**

**Can you tell me at what times in your life this has happened?**

*** Have you ever paid someone to have sex with him or her?**

**Can you tell me at what times in your life this has happened?**

*** Have you ever been forced to have sex against your will?**

Q14. Has this forced sex happened several times with the same person?

1. Yes

2. No

Chap4

**We are now going to talk about children and your pregnancies**

1. FEMME

Q16. Have you had children?

1. Yes

2. No

| **- When was your first child born?**  **- Did you want a child at that time?**  1. You did not ask yourself this question  2. Not at all  3. You wanted a child but later  4. You wanted a child but earlier  5. Yes, at the time  **- When the pregnancy began, were you or your partner doing something to prevent a pregnancy?**  1. Yes  2. No  **- Where does this child live currently?**  1. In France with you  2. In France but not with you  3. In my home country  4. In another country  5. The child died  6. Other  **- Have you had other children, whether with the same person or another person?**  **And when was your second child born?** |
| --- |

**Now, let’s talk about all the other times you were pregnant but the pregnancy did not end by a birth.**

**Q16bis. Has this ever happened to you?**

1. Yes

2. No

| In what year did the first pregnancy of this type happen?  - Was this an induced abortion, spontaneous abortion (or miscarriage) or stillbirth?  **- At the time this pregnancy began, did you want to get pregnant?**  1. You did not ask yourself this question  2. Not at all  3. You wanted a child but later  4. You wanted a child but earlier  5. Yes, at the time  **- When the pregnancy began, were you or your partner doing something to prevent a pregnancy?**  1. Yes  2. No  Have you had another pregnancy that did not end with a birth?  What year did this pregnancy happen? |
| --- |

1. HOMME

We will now talk about children and your partner’s pregnancies

Q16. Have you had children of your own?

1. Yes

2. No

| **When was your first child born?**  **- Did you want a child at that time?**  1. You did not ask yourself this question  2. Not at all  3. You wanted your partner to be pregnant but later  4. You wanted your partner to be pregnant but earlier  5. Yes, at the time  **- When the pregnancy began, were you or your partner doing something to prevent a pregnancy?**  1. Yes  2. No  **- Where does this child live currently?**  1. In France with you  2. In France but not with you  3. In my home country  4. In another country  5. The child died  6. Other  **- Have you had other children, whether with the same person or another person?**  **And when was your second child born?** |
| --- |

**Now, let’s talk about all the other times one of your partners were pregnant but the pregnancy did not end by a birth.**

**Q16bis. Has this ever happened to you?**

1. Yes

2. No

| In what year did the first pregnancy of this type happen?  - Was it an induced abortion, spontaneous abortion (or miscarriage) or stillbirth?  **- At the time this pregnancy began, did you want your partner to get pregnant?**  1. You did not ask yourself this question  2. Not at all  3. You wanted your partner to be pregnant but later  4. You wanted your partner to be pregnant but earlier  5. Yes, at the time  **- When the pregnancy began, were you or your partner doing something to prevent a pregnancy?**  1. Yes  2. No  Has one of your partners had another pregnancy that did not end with a birth?  What year did this pregnancy happen? |
| --- |

Chap5

**We will now discuss another subject**

**As this study is particularly interested in two important diseases that are HIV/AIDS and hepatitis B, we will now try to identify the screening tests you may have done for these two diseases in your life.**

**Let us first talk about hepatitis B testing:**

**Q18. Have you ever been tested for hepatitis B?**

1. Yes

2. No

| **Can we now see at what point in your life you were tested for hepatitis B?**  **- In what year did you take your first test for hepatitis B, and what was its outcome?**  **- On what occasion was this test done?**  1. during a health check  2. because the doctor suggested it  3. during a hospitalization  4. during a blood donation  5. during pre-marital testing  6. during prenatal testing  7. to obtain your residence papers  8. to take a loan, get a job, travel to another country  9. because you were sick (including STIs - Sexually Transmitted Infections)  10. because you wanted to know  11. because someone you know had hepatitis B  12. because you took a risk  13. because you wanted to stop condom use  14. because you wanted to get vaccinated  15. for another reason ……………………. specify  **- Did you then take another test?**  **- On what occasion ...?**  **What year did you first consult a doctor for this disease?**  **Have you ever had a medical follow–up since this consultation, or have you been more than 12 months without seeing your doctor?** |
| --- |

**Q19. Ego reported at least one positive test for hepatitis B**

1. Yes

2. Yes, but the doctor then said that hepatitis was cured

3. No

**Q20. Has someone you know been informed that you have hepatitis B?**

1. Yes you did it yourself

2. Yes someone else did with your consent

3. Yes someone else did without your consent

4 – No

**Q22. Who was informed first?**

1. Your spouse

2. Your children

3. Your father

4. Your mother

5. The father or mother of your spouse or former spouse

6. Your sister(s)

7. Your brother(s)

8. Another family member

9. Friend(s)

10. Neighbour(s)

11. Colleague(s)

12. A religious leader

13. Another person, specify ...............

**Q21. After how long?**

|___|___| Days / Weeks / Months / Years

**Let’s get back to the relationships we spoke about:**

**For ...,**

**701. When you were together, did you know you had hepatitis B?**

1. Yes

2. No

**702. And did X know that you had hepatitis B?**

1. Yes

2. No

3. Doesn’t know

**703. And did X have hepatitis B?**

1. Yes

2. No

3. Doesn’t know

**Q23. Have you been immunised against hepatitis B?**

1. Yes

2. No

Now lets discuss about the tests you may have done for HIV:

Q24. Have you ever been tested for the AIDS virus (HIV)?

1. Yes

2. No

| **Can we now see at what point in your life you were tested for HIV?**  **- In what year did you take your first test for HIV, and what was its outcome?**  **- On what occasion was this test done?**  1. during a health check  2. because the doctor suggested it  3. during hospitalization  4. during a blood donation  5. during pre-marital testing  6. during prenatal testing  7. to obtain your residence papers  8. to take a loan, get a job, travel to another country  9. because you were sick (including STI - Sexually Transmitted Infections)  10. because you wanted to know  11. because your partner was infected  12. because you took a risk  13. because you wanted to stop condom use  14. because the condom ruptured or was mishandled  15. for another reason ……………………. specify  **- Did you then take another test?**  **- On what occasion ...?**  **What year did you first consult a doctor for this disease?**  **Have you ever had a medical follow–up since this consultation, or have you been more than 12 months without seeing your doctor?**  **Do you have an idea of the period during which you were infected by HIV?** |
| --- |

**Q25. Ego reported at least one positive test for HIV**

1. Yes

2. No

**Q26. Why do you think that you were infected at that time?**

**Q27. Has someone you know been informed that you have HIV?**

1. Yes you did it yourself

2. Yes someone else did with your consent

3. Yes someone else did without your consent

4. No

**Q28. Who was informed first?**

1. Your spouse

2. Your children

3. Your father

4. Your mother

5. The father or mother of your spouse or former spouse

6. Your sister(s)

7. Your brother(s)

8. Another family member

9. Friend(s)

10. Neighbour(s)

11. Colleague(s)

12. A religious leader

13. Another person, specify ...............

**Q29. After how long?**

|___|___| Days / Weeks / Months / Years

**Let’s get back to the relationships we spoke about:**

**For ...,**

**701. When you were together, did you know that you were HIV-infected?**

1. Yes

2. No

**702bis. How did X react when he/she learned about your HIV status?**

□ He/she was sympathetic and helped you

□ He/she has become more distant

□ He/she was angry and shouted

□ He/she was violent and hit you

□ He/she left you

□ No answer

□ Other

If other: specify: ............................................

**703. And was X infected with HIV?**

1. Yes

2. No

3. Doesn’t know

Chap6

We will now talk about your overall health.

Q30. How many visits have you attended [show if a hospital] "in this unit" / [show if health centre] "in this centre" in the last 12 months including today | ___ | ___ |

Q30a. Would you say

1. This is the first time

2. Once a year

3. Twice a year

4. Every three months

5. Once a month

6. More often

**Q31. Have you notified your referent doctor to the health care insurance?**

1. Yes

2. No

3. Doesn’t know

97. Not applicable

**Q32. This doctor is:**

1. A general practitioner in a private practice

2. A general practitioner in a health centre

3. The physician who follows you for HIV in hospital

4. Another doctor

**Q32. This doctor is:**

1. A general practitioner in a private practice

2. A general practitioner in a health centre

3. The physician who follows you for hepatitis B in hospital

4. Another doctor

**Q32. This doctor is:**

1. A general practitioner in a private practice

2. A general practitioner in a health centre

3. Another doctor

**Q33. At the moment, how would you qualify your overall health status?**

1. Very good

2. Good

3. Average

4. Bad

5. Very bad

**Q34. Over the past 2 weeks, how often have you been bothered by the following problems?**

1. A feeling of nervousness, anxiety or tension (*Never / several days / More than half of the time / almost every day*)

2. An inability to stop worrying or to control your own worries (*Never / several days / More than half of the time / almost every day*)

3. Little interest or pleasure in doing things (*Never / several days / More than half of the time / almost every day*)

4. Being sad, depressed or hopeless (*Never / several days / More than half the time / almost every day*)

**Q35a. Outside your hepatitis B, do you currently have any other chronic disease, that is to say, a disease that lasts or reoccurs regularly?**

1. Yes

2. No

**Q35a1. What chronic disease(s)?**

**Q35b. Outside of your HIV infection, do you currently have any other chronic disease, that is to say, a disease that lasts or reoccurs regularly?**

1. Yes

2. No

**Q35b1. What chronic disease(s)?**

**Q35c. Outside of your hepatitis B and your HIV infection, do you currently have any other chronic disease, that is to say, a disease that lasts or reoccurs regularly?**

1. Yes

2. No

**Q35c1. What chronic disease(s)?**

**Q35d. Do you currently have a chronic disease, that is to say, a disease that lasts or reoccurs regularly?**

1. Yes

2. No

**Q35d1. What chronic disease(s)?**

**Q36. Have you been limited in everyday activities for at least 6 months due to health problems?**

1. Yes, strongly limited

2. Yes, limited but not strongly

3. Not limited at all

**Q37. Have you ever had another major health problem that has constrained you in your everyday life? (including an accident, serious illness or psychological disturbance)?**

1. Yes

2. No

Q38. Have you ever had tuberculosis?

1. Yes

2. No

**Can you tell me, for each major health problem that you have told me about, at what moment in your life they happened?**

**Q39. Since arriving in France, have you ever been hospitalized for more than a week?**

1. Yes

2. No

Q40. How many times? |___|___|times

| **Can you tell me the year or years in which these hospitalisations occurred?** |
| --- |

**Q41. Do you smoke, be it from time to time?**

1. Yes

2. No

**Q42. You currently smoke**

1. Each day

2. Less often

**Q43. On average, how many cigarettes do you smoke per day?**

1. Fewer than 20 cigarettes

2. More than 20 cigarettes

3. Other (cigars, pipe etc.).

**Q44. How often do you drink alcohol?**

1. You never drink alcohol

2. Once a month or less

3. 2 to 4 times per month

4. 2 to 3 times per week

5. At least 4 times a week

**Q45. How many drinks containing alcohol do you consume on a typical day when you drink?**

1. 1 or 2

2. 3 or 4

3. 5 or 6

4. 7, 8 or 9

5. 10 or more

**Q46. How often do you drink six or more drinks on one special occasion?**

1. Never

2. Less than once per month

3. Once a month

4. Once a week

5. Daily or almost

Chap7

**We will now talk about the time when you arrived in France:**

**Q47. What year did you arrive in France?**

| ___ | ___ | ___ | ___ | (Year) in the month of | ___ | ___ | (Month)

**Q48. Why did you come to France (at that time)?**

1. Join a spouse or fiance(e)

2. Join your children

3. Join another family member (excluding spouse and children)

4. Get married

5. Study

6. Look for work

7. Take a job already identified

8. Try your luck

9. You were threatened in your country

10. For medical reasons

11. Because you came with your parents

12. Do not know/No answer

13. For another reason

**Q48a. Can you tell me what this other reason is?**

**Q49. With whom did you arrive in France?**

ο 1. With spouse

ο 2. With child(ren)

ο 3. With relatives (father, mother, siblings)

ο 4. Alone

ο 5. With another person

**Q49a. Can you tell me who were these people?**

**Q50. At that point, did you already know people who lived in France?**

1. Yes, someone or some people in your family

2. Yes, a spouse or fiancé

3. Yes, one or more friends or acquaintances

4. No

**Q51. If you already knew someone, would you say that ...**

1. At least one of these people helped you

2. At least one of these people did not help you when you asked him/her for help

3. You did not ask for any help.

**Let’s talk now about your nationality.**

**When you were born, what was/were your nationality/ies?**

**Has this changed later on?**

**Can you tell me when?**

**The issue of papers can influence the possibilities that one has to live and work in a country.**

**That is why I would like to try and trace with you the different types of papers, residence permits, you may have had or not living in France. Can we try and trace all that together?**

**When you arrived in France, what administrative document did you have?**

Visa / no visa / no visa requirement / Other

**And then what kind of document did you get?**

Temporary residence authorisation or receipt: TS <1 year

Temporary residence permit: TS 1-3 years

Residence permit: 10 years TS

No residence permit: No TS

No need for a residence permit: No need TS

Other => Specify

**And then did your situation change?**

**What year did it change? What type of document did you get then?**

**And then, did your situation changed... ?**

**Q52. Have you ever applied for a residence permit for a medical reason?**

1. Yes

2. No

**Q53. Did you ever get a residence permit for medical reasons?**

1. Yes

2. No

CHAP8

**Q54. Since your (first) arrival in France, did you have health care insurance coverage: social security, or CMU (Universal Health Coverage) or AME (State Medical Assistance) ...?**

1. Yes

2. No

**From what year did you have health care insurance coverage?**

**Subsequently, have there been periods of at least one year when you no longer had health care insurance coverage?**

**From when to when was that? ...**

**"So currently you do not have health care insurance coverage?"**

**Q55. And now what type of health care insurance coverage do you have?**

1. Social Security

2. CMU (Universal Health Coverage)

3. AME (State Medical Assistance)

4. No health insurance

5. Other

**Q55a. If «other», specify:……………………………………………**

**Q56. Do you now have a complementary/private health care insurance?**

1. Yes

2. No

**Q57. Do you currently have 100% health care insurance coverage for long-term illness?**

1. Yes

2. No

**We will now discuss the problems you have encountered in healthcare facilities:**

**Q58. Since you arrived in France, have you ever NOT been to consult a doctor or dentist whereas you needed it at that time?**

1. Yes once

2. Yes several times

3. No

**Q59. Why has this happened to you ...**

1. You could not pay for the consultation?

2. You preferred to wait and get better?

3. You did not know who to see?

4. Because of language problems?

5. Because of problems with your papers?

6. You were afraid of care?

7. You did not have time to go to the doctor?

8. It was too far or too complicated to go?

9. For another reason

**Q59a. If 'other', please explain: ____________________________________________________________**

**Q60. Have you ever been denied care or drug delivery?**

a) At a doctors (Yes - No - No response)

b) At the hospital (Yes - No - No response)

c) At a pharmacy (Yes-No-No Answer)

Q61. In your opinion, what was related to the fact that you were denied care?

ο 1. Your gender (being male or female)

ο 2. Your health or disability

ο 3. Your skin colour

ο 4. Your origin or your nationality

ο 5. The way you dress

ο 6. The place where you live, your neighbourhood’s reputation

ο 7. Because you're at CMU or AME (for care)

ο 8. Because you have HIV (See if Q25 == 1)

ο 9. Your sexual orientation

ο 10. Your present or past use of alcohol or drugs

ο 11. Your difficulty expressing yourself in French

ο 12. Another reason

ο 98. No answer

Q61a. If 'Another reason', please explain: ________________________________________________________

Chap9

**I will now ask more specific questions about your health, especially related to sexuality. Again, some questions may not apply to you and you just have to say so if this is the case.**

**Q62. How old were you the first time you had sex? | ___ | ___ | Years**

**Q63. Your first sexual intercourse was something:**

1. That you wanted at that time

2. That you accepted but did not really want

3. That you have been forced to do against your will

**Q64. During this first sexual intercourse, did you or your partner do something to prevent a pregnancy or sexually transmitted infections/diseases such as AIDS?**

1. Yes

2. No

**Q65. If yes, what method did you use?**

1. Male condom

2. Withdrawal of partner before ejaculation

3. Spermicidal creams, pessaries, sponge

4. Female condom

5. Pill

6. Morning-after pills or emergency contraception

7. No method

8. Other

98. No answer

**Q65a. If 'Other', specify: ……………….**

**Q65b. Was a condom used during this first sexual intercourse?**

1. Yes

2. No

3. Doesn’t know

**There are several methods to prevent a pregnancy. We'll see now see together all those you may have used before coming to France and since you are in France.**

**Q66. Before coming to France, did you or your partner use a method to prevent a pregnancy?**

1. Yes

2. No

**Q67. Before coming to France, what r method(s) did you use?**

1. Pill

2. IUD

3. Injection

4. Contraceptive implant

5. Male condom

6. Female condom

10. Morning-after pill

11. Withdrawal of the partner before ejaculation

12. Avoid sex on days at risk

7. Tubal Ligation – Sterilization

8. Vasectomy

9. Spermicidal creams, suppositories, sponges, rings

13. Other method

Q67a. If «other method», specify:__________________________________________________________

**Q66. Since you arrived in France, did you or your partner use a method to prevent a pregnancy?**

1. Yes

2. No

**Q69. Since you arrived in France, what method(s) did you use?**

1. Pill

2. IUD

3. Injection

4. Contraceptive implant

5. Male condom

6. Female condom

10. Morning-after pill

11. Withdrawal of the partner before ejaculation

12. Avoid sex on days at risk

7. Tubal Ligation – Sterilization

8. Vasectomy

9. Spermicidal creams, suppositories, sponges, rings

13. Other method

Q69a. If «other method», specify:__________________________________________________________

**Q70. Are you circumcised/cut?**

1. Yes

2. No

**CHAP10**

**We will now talk more specifically about the last 12 months:**

**Q100. How many men have you had sex with during the last 12 months / And how many women? / __ / __ / □ No □ Do not know answer**

**Q100a. How many women have you had sex during the last 12 months / And with how many men? / __ / __ / □ No □ Do not know answer**

**Q101. Over the last twelve months, have you happened NOT to have sex for 3 months or more?**

1. Yes

2. No

**Q102. Over the last 12 months, was there a time when you had several sexual partners at the same time?**

1. Yes

2. No

**Q103. Over the last twelve months, you used condoms ...**

1. Systematically with all partners

2. Systematically with some but not with others

3. From time to time with all partners

4. From time to time with some but not with others

5. Never

**We will now talk about the last person you had sex with:**

**Q104. Is it a person with whom you are having or have had in the past, a relationship that lasted over a year?**

1. Yes

2. No

**Q106. At first sex with that person, did you use a condom?**

1. Yes

2. No

**Q107. In general, regarding condom use with this partner during your relationship, you would say:**

1. You never used any condom

2. You used condoms during each intercourse

3. You used condoms occasionally

4. You used condoms and then stopped

5. Doesn’t know

**Q107a. When you stopped using condoms….:**

a. Were you or your partner using a contraceptive

b. Had you taken an HIV test

c. Had your partner taken an HIV test

1 Yes

2. No

3. Doesn’t know

**Q109. Are you with this person right now?**

1. Yes

2. No

**Q110. How long have you been in this relationship?**

1. Less than a week

2. Between one week and one month

3. Between one and six months

4. Between 6 months and one year

**Q111. How long did this relationship last?**

1. It was a one-night stand

2. Less than a month

3. Between one and six months

4. Between 6 months and one year

**Q112. Is/was he/she younger, older or the same age as you?**

1. Younger

2. Older

3. About the same age (+/- 2 years)

4. No answer

**Q112a. How much younger/older (in years)? / __ / __ / Years**

**Q113. Is/was this partner .... :**

1. A man

2. A woman

**Q114. What was his/her education level at the beginning of your relationship?**

1. No schooling

2. Primary School

3. Secondary education (college, high school)

4. Higher education

5. Doesn’t know

**Q115. In which country this person was he/she born? / ______________ /**

**Q116. Do/did you live together?**

1. Yes

2. No

3. No but we plan to live together

**Q117. At first sex with that person, did you use a condom?**

1. Yes

2. No

**Q118. In general, about the condom with this partner in this relationship, you would say:**

1. You never used any condom

2. You used condoms during each intercourse

3. You used condoms occasionally

4 You used condoms and then stopped

**Q118a. When you stopped using condoms….:**

a. Were you or your partner using a contraceptive

b. Had you taken an HIV test

c. Had your partner taken an HIV test

1 Yes

2. No.

3. Doesn’t know

**Q121avih. Does he/she know that you have HIV?**

1. Yes

2. No

- **How did X react when he/she learned about your HIV status?**

□ He/she was sympathetic and helped you

□ He/she has become more distant

□ He/she was angry and shouted

□ He/she was violent (e) and hit you

□ He/she left you

□ No answer

□ Other

**Q121a2VIH. Is she/he HIV positive?**

1. Yes

2. No

3. Doesn’t know

**Q121a3VIH. When you were together, did you know that you were HIV positive?**

1. Yes

2. No

**Q121a4VIH. Did she/he know that you were HIV positive?**

1. Yes

2. No

3. Doesn’t know

**Q121a4VIHbis. How did X react when he/she learned about your HIV status?**

□ He/she was sympathetic and helped you

□ He/she has become more distant

□ He/she was angry and shouted

□ He/she was violent (e) and hit you

□ He/she left you

□ No answer

□ Other

**If other, specify: …………………………**

**Q121a5VIH. Was she/he HIV positive?**

1. Yes

2. No

**Q121ahepB. Does he/she know that you have hepatitis B?**

1. Yes

2. No

3. Refused

**Q121a1hepB. Does he/she have hepatitis B?**

1. Yes

2. No

**Q121a2hepB. When you were together, did you know you had hepatitis B?**

1. Yes

2. No

**Q121a3hepB. Did he/she know that you had hepatitis B?**

1. Yes

2. No

3. Refused

**Q121a4hepB. And, did he/she have hepatitis B?**

1. Yes

2. No

**Q122. Are you currently using a method to prevent a pregnancy?**

1. Yes

2. No

**Q123. Which method(s) are you using?**

1. Pill

2. IUD

3. Injection

4. Contraceptive implant

5. Male condom

6. Female condom

7. Tubal Ligation – Sterilization

8. Vasectomy

9. Spermicidal creams, suppositories, sponges, rings

10. Morning-after pill

11. Other method

If «other method», specify:__________________________________________________________

**Q124. You are not currently using any contraceptie. Is it because ...?**

1. You are not having sex

2. You want to have a child

3. You have just given birth or are breastfeeding

4. You avoid intercourse on days at risk or you use condoms or withdrawal

5. No contraceptive method works for you or your partner

6. Other situation (menopause, homosexual ...)

**If 'other situation', please explain: _______________________________________________________________**

**Q125. Currently, are you very satisfied, somewhat satisfied, or not at all satisfied with your current sex life?**

1. Very satisfied

2. Fairly satisfied

3. Dissatisfied

Chap10

We will now talk about the people close to you, and first about the people you live with.

Q126. Today, are you living with someone?

1. Yes

2. No. I live alone

We will now list the people with whom you are currently living.

Q127. The .............. person is ...?

1. A man

2. A woman

Q128. How old is he/she?

1. 0 to 12 years

2. Between 13 and 17 years

3. 18 years and over

Q129. What is your relationship with this person? This person is he/she?

1. Spouse/Partner

2. Son/daughter

3. Mother/Father

4. Sister/Brother

5. Niece/Nephew

6. Aunt/Uncle

7. Grandfather/grandmother

8. Other

Q130. Is he/she:

1. At school/Student

2. At work

3. At home/unemployed

**Q131. Has this person ever been tested for hepatitis B?**

1. Yes

2. No

3. Don’t know

**Q132. Has he/she been immunised against hepatitis B?**

1. Yes

2. No

3. Don’t know

**Q133. Do you live with someone else?**

1. Yes

2. No

**Q134. In the dwelling you live in, how many rooms besides the kitchen, shower and toilet are there? | __ | __ |**

97. No housing

**Q143. What is the language you speak most often at home (where you live)?**

⬜ 1. Language of the parents/region of origin

⬜ 2. French

⬜ 3. English

⬜ 4. Other .................................

**Q144. What is the language you speak most often with your friends?**

⬜ 1. Language of the parents/region of origin

⬜ 2. French

⬜ 3. English

⬜ 4. Other .................................

**Q145. Do you have someone close to you with whom you can celebrate good news, a happy event?**

1. Yes in France

2. Yes in the country of origin

3. no

**Q147. Currently, do you have someone close to you can rely on in times of hardship?**

1. Yes in France

2. Yes in the country of origin

3. no

**Q148. Who is that person?**

1. Your spouse

2. Your child(ren)

3. Your father

4. Your mother

5. The father or mother of your spouse or former spouse

6. Your sister(s) or your brother(s)

7. Another family member

8. Friend(s)

9. Neighbour(s)

10. Colleague(s)

11. A member of an association

12. A caregiver

13. A religious leader

14. Another person, specify ...............

**If “other”, specify…………………..**

**Q140. What is your religion?**

1. Catholic

2. Protestant

3. Evangelist

4. Jewish

5. Muslim

6. No religion

8. Another religion

If 'Another religion', specify: _______________________________________________

**Q141. With respect to religion, would you say that you…..:**

1. Do not practice

2. Practice from time to time

3. Practice only when important events (weddings, baptisms, funerals, etc..)

4. Practice regularly

**Apart from doctors/caregivers, who are the people who currently know about your HIV status?**

|  | **Q155** in France | **Q156** in your country of origin | **Q157** in another country |
| --- | --- | --- | --- |
| 1. Your spouse |  |  |  |
| 2. Your child(ren) |  |  |  |
| 3. Your father |  |  |  |
| 4. Your mother |  |  |  |
| 5. Your sister(s) |  |  |  |
| 6. Your brother(s) |  |  |  |
| 7. Another member of the family |  |  |  |
| 8. A friend |  |  |  |
| 9. A social worker, an association, or a professional |  |  |  |
| 10. Another person |  |  |  |
| 11. No one knows |  |  |  |
| 98. No response |  |  |  |

Q158. Overall, have the people close to you changed their attitude towards you since they are aware of your HIV status?

1. Yes, they are closer to you

2. Yes, they are more distant

3. No, nothing has changed

4. I don’t know

5. Other

If 'Other', specify: _______________________________________________

Q159. Has your attitude changed towards the people close to you since your HIV diagnosis?

1. Yes, you took your distance with some people

2. Yes, you are closer to some people

3. No, nothing has changed

4. I don’t know

**Apart from doctors/caregivers, who are the people who currently know about your hepatitis B?**

|  | **Q160** in France | **Q161** in your country of origin | **Q162** in another country |
| --- | --- | --- | --- |
| 1. Your spouse |  |  |  |
| 2. Your child(ren) |  |  |  |
| 3. Your father |  |  |  |
| 4. Your mother |  |  |  |
| 5. Your sister(s) |  |  |  |
| 6. Your brother(s) |  |  |  |
| 7. Another member of the family |  |  |  |
| 8. A friend |  |  |  |
| 9. A social worker, an association, or a professional |  |  |  |
| 10. Another person |  |  |  |
| 11. No one knows |  |  |  |
| 98. No response |  |  |  |

Q163. Overall, have the people close to you changed their attitude towards you since they are aware of your Hepatitis B?

1. Yes, they are closer to you

2. Yes, they are more distant

3. No, nothing has changed

4. I don’t know

5. Other

If 'Other', specify: _______________________________________________

Q164. Has your attitude changed towards the people close to you since your hepatitis B diagnosis?

1. Yes, you took your distance with some people

2. Yes, you are closer to some people

3. No, nothing has changed

4. I don’t know

In France today, what would you say about the following situations and activities?

|  | Fine | Difficult | Not concerned | No response |
| --- | --- | --- | --- | --- |
| **Q149.** As part of your job or your job search (relationship with colleagues and superiors, employment centre procedures…) |  |  |  |  |
| **Q151**. For medical activities, make an appointment, know where to go, update medical coverage…. |  |  |  |  |
| **Q152**. To move around, take the subway, know your bearings in public transport |  |  |  |  |
|  |  |  |  |  |

Q169. Overall, using the images below, can you tell me how you feel in France:

1. I do not feel well at all

2. I feel rather badly

3. I do not feel either good or bad

4. I'm feeling pretty good

5. I feel perfectly fine

Q170. Currently, what are the projects you have at heart and want to achieve?

____________________________________________________________________________________________________________________

**Now, by using the grid that we have completed together, could you show me how you would divide your life?**

**1. From a general point of view, could you divide your life in different periods according on whether you consider these periods as being very good years, good years, neither good or bad years, difficult years, very difficult years**

"TB" if a very good year

"B" if the good years

“NN" if neither good nor bad

"D" if the difficult years

"TD" if very difficult years

**2. Are there personal events (happy or unhappy) or historical events that have influenced your life? Which ones?**

The interview is now complete. Thank you for answering my questions.
