# Supplementary material for: Dynamic of HIV-testing after arrival in France for migrants from sub-Saharan Africa: The role of both health and social care systems
Source: PLoS One. 2017 Dec 21;12(12):e0188751. doi: 10.1371/journal.pone.0188751 (PMC5739385; doi:10.1371/journal.pone.0188751)
Supplement: S2 Appendix — (PDF) [file pone.0188751.s002.pdf]

ENQUETE BIOGRAPHIQUE
"MIGRANTS, PARCOURS DE VIE, PARCOURS DE SANTE"

GRILLE BIOGRAPHIQUE

NOM DE L'ENQUETEUR : ....

N° DE L'ENQUETEUR : |___|___| |___|___|

N° D'INTER CAPI : |___|___| |___|___|

DATE DE L'ENTRETIEN : |___|___| |___|___| |___|___|___|___|

N° D'ANONYMAT : |___|___|___| |___|___| |___|___| |___|___|

ANNÉES	AGES	1 - HISTORIQUE RESIDENTIEL						
			Logement en France					
		**1.1** PAYS	**1.2** DEPARTEMENT	**1.3** Nature du logement	**1.4** Instabilité résidentielle *Cocher la colonne*			
				1 - c'était votre propre logement<br>2 - vous étiez hébergé par votre famille<br>3 - vous étiez hébergé par d'autre personne de votre entourage<br>4 - autre à préciser	Hébergé par des associations ou institutions	Hébergé chez des amis ou familles successifs	Il vous est arrivé de dormir dans un squat	Il vous est arrivé de dormir dans la rue
2012								
2011								
2010								
2009								
2008								
2007								
2006								
2005								
2004								
2003								
2002								
2001								
2000								
1999								
1998								
1997								
1996								
1995								
1994								
1993								
1992								
1991								
1990								
1989								
1988								
1987								
1986								
1985								
1984								
1983								
1982								
1981								
1980								
1979								
1978								
1977								
1976								
1975								
1974								
1973								
1972								
1971								
1970								
1969								
1968								
1967								
1966								
1965								
1964								
1963								
1962								
1961								
1960								
1959								
1958								
1957								
1956								
1955								
1954								
1953								

ANNÉES	9<br>BIEN-ÊTRE	10<br>MOMENTS MARQUANTS
2012		
2011		
2010		
2009		
2008		
2007		
2006		
2005		
2004		
2003		
2002		
2001		
2000		
1999		
1998		
1997		
1996		
1995		
1994		
1993		
1992		
1991		
1990		
1989		
1988		
1987		
1986		
1985		
1984		
1983		
1982		
1981		
1980		
1979		
1978		
1977		
1976		
1975		
1974		
1973		
1972		
1971		
1970		
1969		
1968		
1967		
1966		
1965		
1964		
1963		
1962		
1961		
1960		
1959		
1958		
1957		
1956		
1955		
1954		
1953		

ANNÉES	3 - HISTOIRE DES RELATIONS			4 - GROSSESSES et ENFANTS			
	**3.1**<br>RELATIONS LONGUES (PLUS D'1 AN)	**3.2**<br>RELATIONS COURTES OU OCCASIONNELLES	**3.3**<br>RELATIONS CONTRAINTES OU PAYANTES<br>1 - Transactionnelle (TRANSAC) Oui / Non / NR<br>2 - Payés (PAYES) Oui / Non / NR<br>3 - Recours prostitution (PAYANTS) Oui / Non / NR<br>4 - Rapport forcé (FORCES) Oui / Non / NR	**4.1**<br>Enfants & Grossesses	**4.2**<br>Souhait d'enfant & grossesse<br>1 - Vous ne vous posiez pas la question<br>2 - Pas du tout<br>3 - Vous vouliez un enfant mais plus tard<br>4 - Vous vouliez un enfant mais plus tôt<br>5 - Oui, à ce moment là<br>98 - Non réponse<br>99 - Ne sait plus	**4.3**<br>Au moment où la grossesse a débuté, Contraception<br>1. Oui<br>2. Non	**4.4**<br>Où vit cet enfant actuellement ?<br>1 - En France avec moi<br>2 - En France mais pas avec moi<br>3 - Au pays<br>4 - Dans un autre pays<br>5 - L'enfant est décédé<br>6 - Autre<br>98 - Non réponse<br>99 - Ne sait pas
2012							
2011							
2010							
2009							
2008							
2007							
2006							
2005							
2004							
2003							
2002							
2001							
2000							
1999							
1998							
1997							
1996							
1995							
1994							
1993							
1992							
1991							
1990							
1989							
1988							
1987							
1986							
1985							
1984							
1983							
1982							
1981							
1980							
1979							
1978							
1977							
1976							
1975							
1974							
1973							
1972							
1971							
1970							
1969							
1968							
1967							
1966							
1965							
1964							
1963							
1962							
1961							
1960							
1959							
1958							
1957							
1956							
1955							
1954							
1953							

ANNÉES	REMARQUES ET PRECISIONS
2012	
2011	
2010	
2009	
2008	
2007	
2006	
2005	
2004	
2003	
2002	
2001	
2000	
1999	
1998	
1997	
1996	
1995	
1994	
1993	
1992	
1991	
1990	
1989	
1988	
1987	
1986	
1985	
1984	
1983	
1982	
1981	
1980	
1979	
1978	
1977	
1976	
1975	
1974	
1973	
1972	
1971	
1970	
1969	
1968	
1967	
1966	
1965	
1964	
1963	
1962	
1961	
1960	
1959	
1958	
1957	
1956	
1955	
1954	
1953	

ANNÉES	5 TESTS DE DEPISTAGE		6 HISTOIRE DES MALADIES ET HOSPITALISATION	7 NATIONALITES & TITRES DE SEJOUR	8 PROTECTION MALADIE
	5.1 HEPATITE B	5.2 VIH			
2012					
2011					
2010					
2009					
2008					
2007					
2006					
2005					
2004					
2003					
2002					
2001					
2000					
1999					
1998					
1997					
1996					
1995					
1994					
1993					
1992					
1991					
1990					
1989					
1988					
1987					
1986					
1985					
1984					
1983					
1982					
1981					
1980					
1979					
1978					
1977					
1976					
1975					
1974					
1973					
1972					
1971					
1970					
1969					
1968					
1967					
1966					
1965					
1964					
1963					
1962					
1961					
1960					
1959					
1958					
1957					
1956					
1955					
1954					
1953					

2. HISTORIQUE DES ACTIVITES

ANNÉES	**2.1** Que faisiez-vous...?	SI AU TRAVAIL — **2.2** Etiez-vous : 1- Cadre; 2 - Employé ou ouvrier; 3 - Patron, employeur; 4 - A son compte (sans salariés); 5 - Apprenti, stagiaire; 6 - Aide familial	SI AU TRAVAIL — Activités en France — **2.3** Si salarié, Aviez-vous sur la plus grande partie de la période : 1 - un CDI; 2 - un CDD, Interim; 3 - Pas de contrat	**2.4** D'où venaient vos ressources? *(plusieurs codes possibles pour une même période)* 1 - de votre propre activité; 2 - de l'activité de votre conjoint/partenanire; 3 - d'activités d'autres membres de la famille; 4 - d'allocations de l'état (allocation familiale, allocation de handicap ou invalidité...) : A préciser; 5 - aucune ressource
2012				
2011				
2010				
2009				
2008				
2007				
2006				
2005				
2004				
2003				
2002				
2001				
2000				
1999				
1998				
1997				
1996				
1995				
1994				
1993				
1992				
1991				
1990				
1989				
1988				
1987				
1986				
1985				
1984				
1983				
1982				
1981				
1980				
1979				
1978				
1977				
1976				
1975				
1974				
1973				
1972				
1971				
1970				
1969				
1968				
1967				
1966				
1965				
1964				
1963				
1962				
1961				
1960				
1959				
1958				
1957				
1956				
1955				
1954				
1953				
